# Feasibility of radioguided occult lesion localization of clip-marked lymph nodes for tailored axillary treatment in breast cancer patients treated with neoadjuvant systemic therapy

**DOI:** 10.1186/s13550-019-0560-3

**Published:** 2019-10-24

**Authors:** Daan Hellingman, Maarten L. Donswijk, Gonneke A. O. Winter-Warnars, Petra de Koekkoek-Doll, Marilyn Pinas, Yvonne Budde-van Namen, Johan Westerga, Marie-Jeanne T. F. D. Vrancken Peeters, Nikola Kimmings, Marcel P. M. Stokkel

**Affiliations:** 1 Department of Nuclear Medicine, Netherlands Cancer Institute-Antoni van Leeuwenhoek, Postbus 90203, 1006 BE Amsterdam, The Netherlands; 2 Department of Radiology, Netherlands Cancer Institute-Antoni van Leeuwenhoek, Postbus 90203, 1006 BE Amsterdam, The Netherlands; 3 Department of Radiology, Slotervaart hospital, Postbus 90440, 1006 BK Amsterdam, The Netherlands; 4 Department of Radiology, Haaglanden Medical Center, Postbus 432, 2501 CK The Hague, The Netherlands; 5 Department of Pathology, Slotervaart hospital, Postbus 90440, 1006 BK Amsterdam, The Netherlands; 6 Department of Surgical Oncology, Netherlands Cancer Institute-Antoni van Leeuwenhoek, Postbus 90203, 1006 BE Amsterdam, The Netherlands; 7 Department of Surgical Oncology, Slotervaart hospital, Postbus 90440, 1006 BK Amsterdam, The Netherlands; 8 Department of Surgical Oncology, Alexander Monro hospital, Postbus 181, 3720 AD Bilthoven, The Netherlands

**Keywords:** Breast cancer, neoadjuvant systemic treatment, axillary staging, radioguided occult lesion localization, target lymph node technique

## Abstract

**Background:**

Selective removal of initially tumor-positive axillary lymph nodes in breast cancer patients who underwent neoadjuvant systemic treatment (NST) improves the accuracy of nodal staging and provides the opportunity for more tailored axillary treatment. This study evaluated whether radioguided occult lesion localization (ROLL) of clip-marked lymph nodes is feasible in clinical practice.

**Methods:**

Prior to NST, a clip marker was placed inside a proven tumor-positive lymph node in all breast cancer patients (cTis-4N1-3 M0). After NST, technetium-99m-labeled macroaggregated albumin was injected in the clip-marked lymph nodes. The next day, these ROLL-marked nodes were selectively removed at surgery to evaluate the pathological response of the axilla.

**Results:**

Thirty-seven patients (38 axillae) underwent clip insertion. After NST, the clip was visible by ultrasound in 36 procedures (95%). In the other two patients, the ROLL-node injection was performed in a sonographically suspicious unclipped node (1), and near the clip under computed tomography guidance (1). Initial surgery successfully identified the ROLL-marked node with clip in 33 procedures (87%). Removed specimens in the other five procedures contained only the sonographically suspicious tumor-positive unclipped node (1), a node with signs of complete response but no clip (2), a clip without node (1), and tissue without node nor clip, and a second successful ROLL-node procedure was performed (1). Overall, 10 ROLL-marked nodes had no residual disease.

**Conclusions:**

This study demonstrates that the ROLL procedure to identify clip-marked lymph nodes is feasible. This facilitates selective removal at surgery and may tailor axillary treatment in patients treated with NST.

## Background

Neoadjuvant systemic treatment (NST) is applied for tumor downstaging to facilitate breast-conserving therapy in patients with node-negative or node-positive disease [1]. Pathological complete response (pCR) of axillary lymph nodes in patients who were node-positive prior to NST is associated with improved disease-free survival [2] and potentially minimizes the need for axillary lymph node dissection (ALND) [3]. After NST, conversion to a pathological node-negative status occurs in 41–74%, mainly depending on the NST regimen and tumor subtype, of the node-positive patients [4–6].

Although sentinel lymph node (SLN) biopsy has become the standard of care for nodal staging of clinically node-negative breast cancer patients [7], SLN biopsy after NST is less reliable in patients who had tumor-positive axillary nodes prior to NST. Prospective multi-center studies reported false-negative rate (FNR) of > 10% [8–10]. The FNR improved to below 10% only in selected procedures when 3 or more nodes were removed or the dual mapping technique (radioisotope and blue dye) was performed. Therefore, a more accurate nodal staging method is preferred to tailor further axillary treatment.

Caudle et al. developed the targeted axillary dissection (TAD) technique which resulted in a FNR below 5% [11]. The TAD technique involves SLN biopsy as well as radioguided removal of a marked node that was tumor-positive prior to NST. Node marking consists of a two-step procedure. A clip is placed in the biopsy-confirmed metastatic node prior to NST, and subsequent placement of an iodine-125 seed in this marked node is performed after completion of NST within 1–5 days before surgery. Importantly, this study showed that removal of the clipped node alone resulted in a better FNR than performing only SLN biopsy, 4.2% versus 10.1%, respectively.

Simultaneously, a technique solely based on removal of the pre-NST marked node without SLN biopsy was developed, the so-called MARI (marking of the axilla with radioactive iodine-125 seeds) procedure [12]. Dutch regulations allowed this one-step marking procedure in which one of the proven positive nodes at initial diagnosis is immediately marked with an iodine-125 seed and selectively removed after NST. Here, the reported FNR was 7% when isolated tumor cells were counted as positive, and the FNR was below 5% when isolated tumor cells were counted as negative [13].

Although iodine-125 seeds are increasingly being used for breast-conserving surgery and provide excellent surgical guidance in locating axillary lymph nodes [14], there are some practical limitations which hamper their implementation. The use of iodine-125 seeds for this specific indication requires specific radiation safety procedures and documentation to limit the risk of damage or loss of these seeds [15, 16]. Therefore, not all hospitals, or even countries, are authorized to use iodine-125 seeds for locating axillary lymph nodes.

To overcome these limitations, we propose a technique for locating clip-marked lymph nodes without SLN biopsy by using an injection of technetium-99m(Tc-99m)-labeled macroaggregated albumin (MAA) instead of iodine-125 seeds. This is a modification of the MARI procedure by using a two-step node marking procedure. The concept is based on two case series studying the radioguided occult lesion localization (ROLL) procedure for identifying axillary lymph nodes. In these studies, Tc-99m-MAA is injected directly into sonographically suspicious axillary lymph nodes [17, 18]. The practical foreseen advantages are that (1) Tc-99m-MAA is widely used and available; (2) documentation for Tc-99m radiotracers is often already in place, so there is no need to set up complex radiation safety procedures; and (3) for surgeons the technique is practically identical to the SLN biopsy procedure using their standard gamma probes.

The present study assessed the feasibility of ROLL injections into clip-marked lymph nodes that were tumor-positive prior to NST and selectively remove them at surgery after NST in breast cancer patients.

## Methods

### Patient selection

Since February 2015, all female patients with clip-marked cytology-proven node-positive breast cancer in the Slotervaart hospital (SVH) and referred to the Nuclear Medicine Department of the Netherlands Cancer Institute (NCI) were prospectively entered into a database. These patients underwent a Tc-99m-MAA injection in the clip-marked lymph node for response evaluation after NST. This procedure was part of the patient’s standard clinical workup as replacement of the MARI procedure as standard clinical practice, since there was no authorization to work with iodine-125 seeds in the SVH. This dual-center retrospective study was approved by the Institutional Review Board of the NCI.

### Diagnostic imaging before NST

**Fig. 1:**
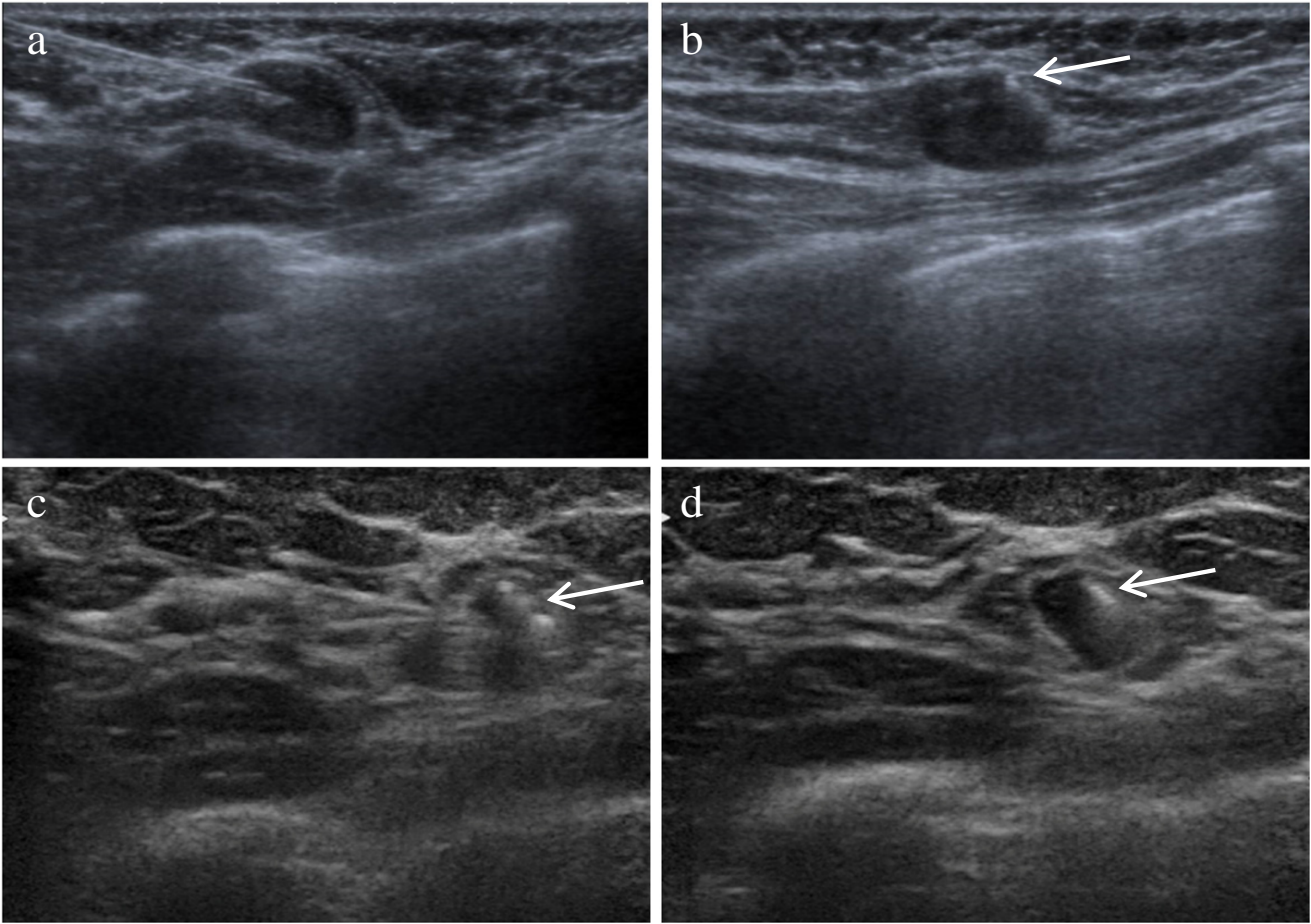
Axillary ultrasound images of a patient with a proven tumor-positive lymph node prior to neoadjuvant systemic treatment (NST). **a** A needle was inserted in the sonographically hypoechoic axillary lymph node to place an O-twistmarker (white arrow) inside (**b**). After NST (**c**), the O-twistmarker was located and a needle was inserted in the lymph node that was no longer suspicious on ultrasound. **d** After injection of 0.2 ml Tc-99m-MAA, a hypoechoic lymph node was observed. Histopathological assessment showed that this node was converted into a node-negative status

Once breast cancer was confirmed, the size and extent of the primary tumor were routinely assessed by mammography, ultrasound, and magnetic resonance imaging (MRI). All patients underwent ultrasound of the breast and axilla. Whole-body [18F]fluorodeoxyglucose (18F-FDG) positron emission tomography/computed tomography (PET/CT) was performed before NST for the detection of regional and distant metastases. In addition, PET/CT of the thorax was performed in prone position and with hanging breasts. Fine-needle aspiration was performed in case of suspicious lymph nodes identified by physical examination, ultrasound, MRI, or PET/CT. When cytological evaluation demonstrated metastasis, a clip marker (O-Twist Marker, BARD GmbH, Karlsruhe, Germany) was placed within the largest tumor-positive lymph node under ultrasound guidance by a breast radiologist at the SVH (Fig. 1).

NST was administered according to institutional guidelines. Two patients received only neoadjuvant endocrine therapy due to old age (over 80 years), highly hormone-sensitive tumors, and co-morbidity.

### ROLL-node technique

On the day before surgery, an experienced breast radiologist at the NCI located the clip-marked lymph node with ultrasound to determine the *yi*N status. Suspicious lymph nodes were defined by a cortex of ≥ 2.3 mm. A 21-G needle was placed percutaneously with the tip in the center of the node (Fig 1). Subsequently, 0.2 ml Tc-99m-MAA (TechneScan LyoMAA, Mallinckrodt Medical, Petten, the Netherlands) was injected intranodally by a nuclear medicine physician (mean dose, 18.5 MBq, range 3.7-37 MBq). CT imaging was used to locate the clip and guide the ROLL-node injection when ultrasound failed to identify the clip-marked lymph node. In patients scheduled for breast-conserving surgery, a ROLL procedure of the primary tumor site was performed in the same session. An aimed dose of 37 MBq Tc-99m-MAA was injected near the biopsy clip of the primary tumor. Anterior and lateral oblique 5-min planar lymphoscintigrams were scheduled after tracer injection (Symbia T; Siemens, Erlangen, Germany). Single-photon emission computed tomography combined with CT (SPECT/CT) was only performed when the planar lymphoscintigraphy showed unexpected drainage patterns.

### Surgery

After completion of NST, removal of the ROLL-marked lymph node and breast surgery were performed in the same surgery in the SVH. A hand-held gamma probe (Navigator GPS; RMD Instruments, Watertown, USA) was used to localize the area of maximal radioactivity allowing the appropriate location of the incision. In patients undergoing a mastectomy without primary breast reconstruction, the incision of the mastectomy was used to dissect towards the ROLL-marked node. A separate incision was made in the other patients. Careful dissection was carried out following the area of maximum radioactivity until the ROLL-marked node was identified and excised. Measurements of the ex vivo specimen and background were performed to confirm the removal of the ROLL-marked node. The excised specimens in patients with four or more 18F-FDG-avid axillary lymph nodes on pretreatment PET/CT were sent to the pathologist for intraoperative frozen section analysis. An ALND was performed when the frozen section came back tumor-positive.

### Pathology examination

Histopathological examination was performed on all harvested lymph nodes. All lymph nodes were fixed in formalin, bisected, sliced at 2-mm intervals, and embedded in paraffin, followed by hematoxylin-eosin and immunohistochemical staining (CAM 5.2; Becton Dickin-son, San Jose, CA, USA). The pathologist confirmed the presence of the clip and lymph nodes in the excised specimens.

### Tailored axillary treatment

Tailored axillary treatment was performed after adapting the previously described protocol in which axillary treatment was based on pretreatment PET/CT prior to NST and pathological status of the clipped node after NST [19, 20]. In summary, four groups were generated for tailored treatment of the axilla after NST. The number of 18F-FDG-avid axillary lymph nodes on the pretreatment PET/CT scan was assessed visually to classify patients as cN<4 (one to three positive axillary lymph nodes) or cN4+ (four or more positive axillary lymph nodes). In axillary cN<4 patients with a tumor-positive clip-marked node received axillary radiotherapy, as did axillary cN4+ patients with a tumor-negative clip-marked node. Both ALND and axillary radiotherapy were performed only in cN4+ patients with a tumor-positive clip-marked node after NST. Axillary treatment was omitted in the axillary cN<4 patients with a tumor-negative clip-marked node after NST.

## Results

In total, 1 bilateral (left and right axilla) and 36 unilateral procedures met the inclusion criteria. The clinicopathologic and treatment characteristics of these 37 patients with 38 ROLL-node procedures are listed in Table 1.

**Table 1:** Patient and tumor characteristics

	*n* (%)
No. of procedures	38
Median age (years)	51 (range 31–83)
cT-stage prior to NST	
Tis	1 (2.6)
1	4 (10.5)
2	23 (60.5)
3	6 (15.8)
4	4 (10.5)
cN-stage prior to NST	
1	23 (60.5)
2	9 (23.7)
3	6 (15.8)
Axillary lymph node stage on pretreatment PET/CT	
cN<4 (< 4 18F-FDG-avid nodes)	26 (68.4)
1	10
2	14
3	2
cN4+ (≥ 4 18F-FDG-avid nodes)	12 (31.6)
Tumor histology	
Ductal carcinoma in situ	1 (2.6)
Ductal carcinoma	33 (86.8)
Lobular carcinoma	3 (7.9)
Other	1 (2.6)
Receptor status	
HR-positive/HER2-negative	28 (73.7)
Triple negative	6 (15.8)
HER2-positive	4 (10.5)
NST regimen	
ddAC	7 (18.4)
ddAC + paclitaxel	21 (55.3)
ddAC + CP	3 (7.9)
CP	2 (5.3)
PTC-P	3 (7.9)
Endocrine	2 (5.3)

*Tis* ductal carcinoma in situ, *18F-FDG* [18F]fluorodeoxyglucose, *PET/CT* positron emission tomography combined with computed tomography, *HR* hormone receptor, *HER2* human epidermal growth factor receptor 2, *NST* neoadjuvant systemic treatment, *ddAC* doxorubicine and cyclophosphamide (dose dense), *CP* carboplatin and paclitaxel, *PTC-P* paclitaxel, trastuzumab, carboplatin, and pertuzumab

In 26 procedures (68%), one to three 18F-FDG-avid axillary nodes were detected on PET/CT, and in 12 procedures (32%), four or more nodes were detected.

### ROLL-node injection and imaging

**Fig. 2:**
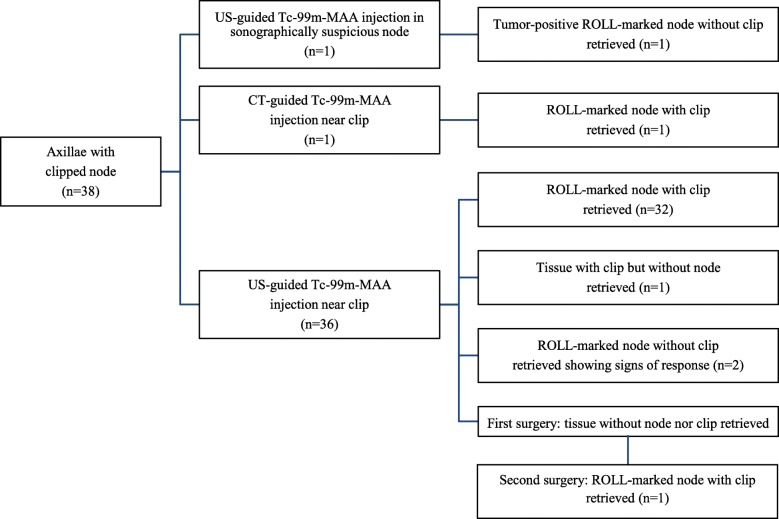
Flowchart of 38 ROLL-node procedures with surgical outcomes. Tc-99m-MAA technetium-99m-labeled macroaggregated albumin, ROLL Radioguided occult lesion localization, US ultrasound, CT computed tomography

In 36 procedures (95%), the ROLL-node injection was performed in close proximity to the clip using ultrasound guidance. The clip could not be visualized sonographically in two patients. Therefore, a sonographically suspicious enlarged unclipped lymph node was injected in one patient and the other patient proceeded to a CT-guided injection of a deeply located clip-marked node (Fig. 2).

**Fig. 3:**
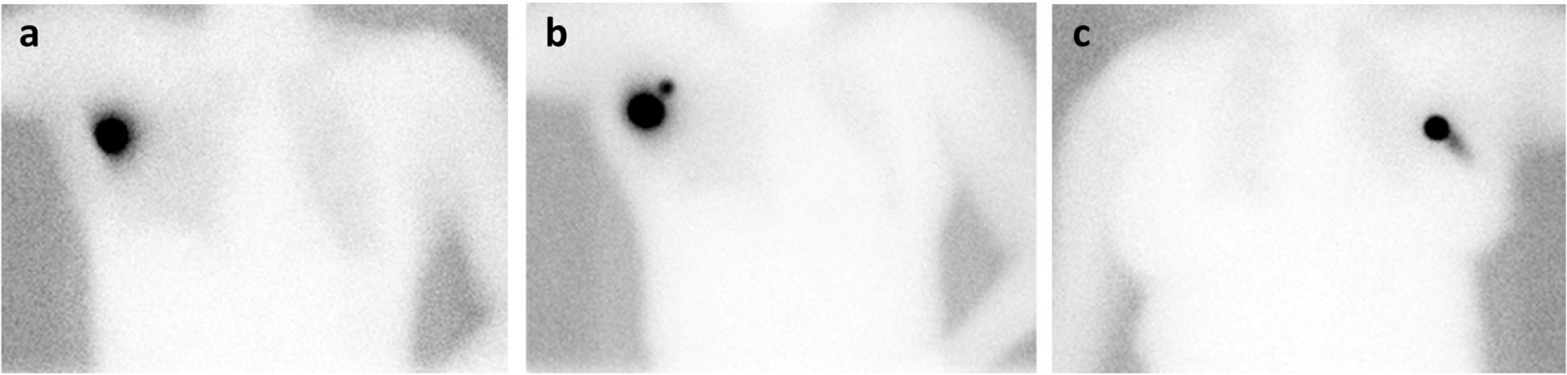
Planar anterior scintigraphy images in three different patients after the ROLL-node injection in the clip-marked lymph node, displaying the three different scintigraphic patterns. **a** One clear focus is observed in the axilla. **b** Two focal hotspots are observed in the axilla, corresponding to the ROLL-marked node and drainage to one higher echelon node. **c** One focal hotspot is observed in the axilla with minimal radioactivity in the needle trajectory.

Scintigraphic images were performed at on average 24 min (range 11–130 min) post-injection. Three different scintigraphic patterns were observed (Fig. 3). Clear, focal activity of the radioactive tracer depot in the axilla was visualized in 23/38 procedures (61%). Additional minimal radioactivity in the needle trajectory was visualized in 10/38 procedures (26%). In 5/38 procedures (13%), drainage to higher echelon nodes was seen. One higher echelon node was visualized in four patients, and two higher echelon nodes were seen in one patient.

Additional SPECT/CT was performed in two patients. SPECT/CT demonstrated that the unexpected second focal hotspot in the axilla was contamination in the needle trajectory in one patient and drainage to a higher echelon node in the other patient.

### Surgery and histopathology

The mean number of retrieved lymph nodes during ROLL-node surgery was 1.5 (range 0–5). Histopathology confirmed that in 95% (36/38) of the procedures, a lymph node was removed. Initial surgery successfully identified the ROLL-marked with clip in 33 of the 37 procedures (89%) in which the Tc-99m-MAA was injected near the clip. A tumor-positive ROLL-marked node was found in the procedure with the injection in the sonographically suspicious unclipped node.

In two patients, only a tumor-negative lymph node was found with signs of response on histopathological assessment. These patients were considered as axillary responders in further tailored axillary treatment despite the missing clip. Post-operative CT confirmed that the clip was still in situ in these two patients.

Both cases in which no lymph node was found were cN<4 patients. The removed specimen of one patient contained only the clip with fatty tissue. This patient was treated as a non-responder and received adjuvant axillary radiotherapy, because the primary tumor showed partial response and pretreatment PET/CT detected also 18F-FDG-avid internal mammary chain nodes. The specimen of the other patient consisted of an old hematoma surrounded by fat without clip. This patient underwent a second ROLL-node procedure of the clip-marked node 3 weeks after the first surgery. This second procedure successfully identified the ROLL-marked node with a clip. Histopathological assessment showed no residual disease, so further axillary treatment was omitted.

Frozen section of the ROLL-marked node was performed in 10 of the 12 axillary cN4+ patients. Additional ALND was performed for 8 of these 10 patients because of a positive frozen section. For two patients, ALND was performed without frozen section since these patients had highly suspicious palpable axillary lymph nodes intraoperatively. In both cases, histopathological results were positive. A median of 7 tumor-positive nodes was found in the 10 ALND specimens (range 2–20).

Overall, 27% (10/37) of the procedures demonstrated a pCR of the ROLL-marked node (Table 2). In 11 procedures (92%) with *yi*N+ and 22 procedures (85%) with *yi*N0 status, the ROLL-marked node with clip was successfully removed during initial surgery.

**Table 2:** Surgery, pathological response, and adjuvant systemic treatment

	*n* (%)
No. of procedures	38
Breast surgery	
Mastectomy	13 (34.2)
Breast-conserving surgery	25 (65.8)
*yp*T-stage	
pCR	7 (18.4)
1	19 (50.0)
2	11 (28.9)
3	1 (2.6)
*yi*N-stage	
*yi*N0	12 (31.6)
*yi*N+	26 (68.4)
*yp*N-stage	
0	10 (26.3)
1	18 (47.4)
2	5 (13.2)
3	4 (10.5)
Unknown^a^	1 (2.6)
Outcome ROLL-marked node	
pCR	10 (26.3)
Marker in node^b^	8
No marker present	2
Residual disease	27 (71.1)
Macro-metastasis	24
Micro-metastasis	2
Isolated tumor cells	1
Unknown^a^	1 (2.6)
Adjuvant systemic therapy	
HT	24 (63.2)
HT + TT	4 (10.5)
HT + CT	1 (2.6)
TT	2 (5.3)
No adjuvant systemic therapy	7 (18.4)

^a^No lymph node was found during the ROLL-node procedure in one patient

^b^In one patient, the ROLL-marked node was initially not found, but removed during a second ROLL-node procedure

*HT* hormone therapy, *TT* targeted therapy, *CT* chemotherapy

### Tailored axillary treatment

**Fig. 4:**
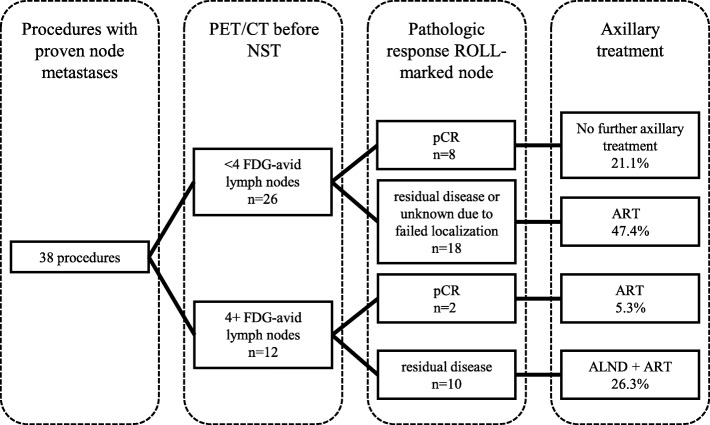
Tailored axillary treatment in patients by combining pretreatment 18F-FDG PET/CT, neoadjuvant systemic treatment (NST), and pathologic response of a ROLL-marked node that was tumor-positive prior to NST. PET/CT positron emission tomography combined with computed tomography, ROLL radioguided occult lesion localization, pCR pathologic complete response, ART axillary radiotherapy, ALND axillary lymph node dissection

Figure 4 shows the tailored axillary treatment of all procedures. Overall, ALND was omitted in 74% (28/38) of the procedures. Axillary radiotherapy was performed in 79% (30/38) of the procedures, and no axillary treatment was given in 21% (8/38) of the procedures. Adjuvant systemic therapy was given in 31 procedures.

No regional recurrences were found in the 10 procedures with a pCR of the ROLL-marked node during a median follow-up of 22 months (range 6–35).

## Discussion

This is the first study that investigated the use of ROLL-node injections into clip-marked lymph nodes that were tumor-positive prior to NST and selectively remove them at surgery after NST. Identifying the ROLL-marked node with clip both preoperatively and at surgery was successful in 87% (33/38) of the procedures. We used a combination of pretreatment 18F-FDG PET/CT and assessment of pathologic complete response of axillary lymph nodes by selectively removing the ROLL-marked node to further tailor axillary treatment, and hereby we have omitted ALND in 74% of our procedures.

There is an emerging interest in selective identification of axillary lymph nodes, that were tumor-positive before NST, to assess pathological response after NST since the SLN procedure after NST in patients with tumor-positive nodes before NST is only successful in a selected subgroup of patients [9]. Since the introduction of iodine-125 seed localization of tumor-positive lymph nodes (the MARI procedure) before NST as target lymph node technique [11, 13], three other localization techniques have been proposed for hospitals that do not have documentation in place for radioactive iodine-125 seed handling and disposal. Choy et al. have described a one-step procedure in which black tattoo ink was injected in suspicious lymph nodes [21]. Ink was injected in a mean number of 1.5 nodes per patient. Patients underwent a standard SLN procedure. Intraoperatively, the black-tattooed node corresponded with the SLN in 96% (27/28) of the patients. The limitation of this technique is that the procedure still involves a SLN procedure. Furthermore, tattoo ink itself cannot be seen nor localized with overlapping tissue, thus extensive tissue handling is necessary to really see the tattooed nodes. Another two-step technique that resembles our ROLL-marked node technique involves the placement of a hookwire into the clip-marked lymph node. The main advantage of the hookwire procedure is that it is a non-radioactive method. However, it requires more complex logistical planning because the hookwire must be placed at the same day of surgery, while ROLL-node injections or iodine-125 seed placement can be scheduled on the day before surgery. Further, hookwire localization is associated with more stress and discomfort for patients in comparison to iodine-125 seed localization and has the risk of dislocation [22]. Finally, intraoperative ultrasound can be used to guide the surgeon in locating the clip-marked node [23].

It is desirable to use definitive markers prior to NST to allow node resection without any other step. In this regard, iodine-125 seeds are still the most suitable markers and currently used in the NCI. A disadvantage of the two-step protocol is that the clip can be difficult to find with ultrasound, especially in nodes that respond well to NST. In our study, the clip was not localized by ultrasound in 2 procedures (5%). Earlier studies have reported that the clip could not be found by ultrasound in 4–28% of the procedures [23–26]. Therefore, it is recommended to have an experienced breast radiologist for the ultrasound-guided procedure and have CT-guidance in place when ultrasound fails to localize the clip.

In two patients, the removed ROLL-marked node showed signs of response but did not contain the clip, although the ROLL-node injection was placed near the clip. Thus, a strict identification of the removed ROLL-marked node being the clip-marked tumor-positive lymph node prior to NST on initial surgery was hampered in these two patients. In a third patient, the removed specimen only contained the clip without any node. These findings suggest that nodal shrinkage during NST may cause clip displacement into the perinodal fatty tissue as was reported by Hartmann et al. [25].

Two limitations of this study were identified. First, the FNR was not evaluated, because ALND was only performed in cN4+;ypN+(ROLL-marked node) patients. Previous studies have proven that marking tumor-positive axillary nodes prior to NST and selectively removing them after NST results in low FNRs [11, 13]. Although not evaluated, the FNR of successful ROLL-node procedures is expected to give similar figures. The primary objective of this study was to report on early learning experiences and challenges to remove ROLL-nodes with clip selectively at surgery. Second, removal of the clip inside the specimen was not confirmed by intraoperative imaging. The surgeon can explore the axilla further with the gamma probe when specimen imaging fails to localize the clip. This may improve the clip-marked node identification rate. We recommend an ALND when the clip cannot be found on specimen imaging or when the clip is located outside the lymph node in order to achieve accurate nodal staging. Most other localization techniques using intraoperative clip removal confirmation reported reasonable to high identification rates. Hookwire localization, radioactive iodine-125 seed localization, or intraoperative ultrasound-guided surgery resulted in intraoperative identification (node with clip) rates of 71–97% [24, 25, 27, 28], 97% [13], or 96% [23], respectively.

Potentially, the ROLL-node procedure can be combined with the SLN procedure. Combining a targeted lymph node procedure with the SLN procedure will reduce the false-negative rate [11]. Lymphoscintigraphy can be used to visualize both the ROLL-marked node and SLNs, although the distinction between the SLNs and the ROLL-marked node is hampered when lymphatic vessels are not visualized. The surgeon can decide to remove all radioactive lymph nodes. Another possibility is using blue dye to identify SLNs. The use of blue dye can identify whether the ROLL-marked node is also a SLN. Preoperative imaging is a valuable tool to visualize unpredictable lymphatic drainage patterns. In this study, drainage to higher echelon nodes was seen in 13% (5/38) of the procedures, despite the use of a relatively large radioactive particle. Preoperative knowledge about the existence of these higher echelon nodes can facilitate the surgical procedure. In our experience, the ROLL-marked node could still be easily distinguished from higher echelon nodes due to knowledge of their respective localization on preoperative lymphoscintigraphy and due to higher radioactive counts at surgery. Larger studies with intraoperative clip removal confirmation are needed to optimize the ROLL-node procedure.

## Conclusions

It is technically feasible to perform ROLL procedures of clip-marked lymph nodes and selectively remove them at surgery. The ROLL-node injection can be performed the day before surgery. This is a promising method for hospitals that cannot use iodine-125 seeds for target lymph node procedures to tailor axillary treatment after NST.

## Data Availability

The datasets used and/or analyzed during the current study are available from the corresponding author on reasonable request.

## Abbreviations

18F-FDG: [18F]fluorodeoxyglucose
ALND: Axillary lymph node dissection
FNR: False-negative rate
MARI: Marking of the axilla with radioactive iodine-125 seeds
MRI: Magnetic resonance imaging
NCI: Netherlands Cancer Institute
NST: Neoadjuvant systemic treatment
pCR: Pathological complete response
PET/CT: Positron emission tomography/computed tomography
ROLL: Radioguided occult lesion localization
SLN: Sentinel lymph node
SVH: Slotervaart hospital
TAD: Targeted axillary dissection
Tc-99m-MAA: Technetium-99m-labeled macroaggregated albumin
